# Immunomodulatory Activity of Complementary and Alternative Medicines 

**DOI:** 10.1155/2014/765107

**Published:** 2014-03-02

**Authors:** Kuzhuvelil Bhaskaran-Nair Harikumar, Roland Hardman, Jesil Mathew Aranjani, Vineesh Vimala Raveendran, Punathil Thejass

**Affiliations:** ^1^Cancer Research Program, Rajiv Gandhi Centre for Biotechnology, Thiruvananthapuram 695014, India; ^2^School of Pharmacy and Pharmacology, University of Bath, Bath BA2 7AY, UK; ^3^Department of Pharmaceutical Biotechnology, Manipal College of Pharmaceutical Sciences, Manipal University, Manipal 576104, India; ^4^Division of Allergy, Clinical Immunology and Rheumatology, University of Kansas Medical Center, Kansas City, KS 66160, USA; ^5^Department of Zoology, Government College, Madappally, Vadakara 673102, India

Around the world, the majority of people tend to adopt different health care approaches outside of the mainstream Western medicine for diseases or general well-being. These nonmainstream health care approaches are commonly referred to as complementary and alternative medicine (CAM).

Although people use the words “complementary and alternative” interchangeably, they refer to two different concepts. “Complementary” confers the intention to add to conventional medicines by the use of phytomedicines, which are nonmainstream medicines. While “alternative” refers to a purely nonmainstream approach using phytomedicines, instead of a combinational medicinal treatment.

The main drawback, raised by the Western medical community, is the lack of scientific evidence for the ascribed healing power of CAM, as well as the failure of large placebo controlled clinical trials to show the therapeutic efficacy of certain CAM. Nevertheless, in certain instances, the research relying on herbal products has led to the discovery of potent anticancer lead molecules such as taxol and camptothecin, and today many other candidate plant molecules are under investigation as anticancer agents. Similarly, plant activity for cardiovascular disease and other inflammatory diseases is relevant. Evidence continues to build up in support of the effects of these therapies on immunomodulation and maintenance of the physiological balance between anti- and proinflammatory mechanisms.

In vitro and in vivo animal studies have shown the plausible mechanisms of CAM in preventing or curing disease to be linked to antioxidant effects, alterations in proinflammatory cell signaling, production of cytokines, and other proinflammatory mediators.

To better understand and adopt these nonmainstream health approaches to complement the current standard (Western) healthcare, scientific evidence is required which is obtained via properly controlled experimentation. In this special issue we have some research papers which hold evidence of efficacy in various disease conditions.

Three papers of this special issue show the effect of either hydrogen dissolved purified water or herbal natural products on atopic dermatitis (AD) like skin inflammation. The authors of one of the articles compared the effect of drinking hydrogen water (HW) or purified water (PW) on AD and showed therapeutic effect by HW over PW by effectively modulating Th1 and Th2 response. In another article, the authors induced AD like inflammation in Balb/c mice by 2, 4-dinitrofluorobenzene and studied the effect of Qingpeng ointment (QP) on alleviation of lesion severity. The authors correlated the effect of QP on AD with the inhibition of infiltration of CD4+T cells and mast cells, suppression of production of IgE and IL-4, and increase in the levels of IFN-*γ*. One of the papers also deals with a natural remedy for AD. The authors used a nona natural product mixture (NPM-9), which effectively suppressed Th2-mediated allergic inflammation on skin of the mouse model.

Another paper attempted to study the effect Korean red ginseng (KRG) on *vif* gene in HIV. Patients receiving KRG and highly active antiretroviral therapy (HAART) exhibited high frequency of premature stop codons (PSC) for *vif* compared to patients on KRG alone or the placebo group. On the other hand, KRG induced more in-frame deletions compared to the placebo group. This study therefore suggests a therapeutic strategy for a better outcome in HIV patients by combining KRG along with HAART.

Another paper demonstrates the effect of a Chinese medicine granule, Shu-Feng-Xuan-Fei (SFXF), on the gross change in the gene expression profile in T-cell-mediated immunity, during influenza virus infection. The authors studied different doses of SFXF and compared the efficacy with the control group as well as with mice which received oseltamivir. The authors concluded that SFXF restored the immune function and helped the rapid clearance of viral particles by regulating the T-cell gene expression patterns.

We believe the above selected research papers, taken from those appearing in this special issue, will improve our knowledge and understanding of the immunomodulatory functions of complementary and alternative medicines.

